# New insights into patterns of first metastatic sites influencing survival of patients with hormone receptor-positive, HER2-negative breast cancer: a multicenter study of 271 patients

**DOI:** 10.1186/s12885-021-08219-3

**Published:** 2021-04-29

**Authors:** Jun Yamamura, Shunji Kamigaki, Junya Fujita, Hiroki Osato, Hironobu Manabe, Yumiko Tanaka, Wataru Shinzaki, Yukihiko Hashimoto, Yoshifumi Komoike

**Affiliations:** 1 Department of Surgery, Sakai City Medical Center, 1-1-1, Ebaraji-cho, Nishi-ku, Sakai City, Osaka 593-8304 Japan; 2 Division of Breast and Endocrine Surgery, Department of Surgery, Kindai University Faculty of Medicine, 377-2, Ohnohigasi, Sayama City, Osaka 589-8511 Japan

**Keywords:** Breast cancer, Recurrence, Survival, Hormone receptor-positive, HER2-negative, Metastatic pattern

## Abstract

**Background:**

The initial therapeutic strategy for hormone receptor-positive (HR+), HER2-negative (HER2-) breast cancer is based on the first metastatic site; however, little evidence is available regarding the influence of metastatic distribution patterns of first metastatic sites on prognosis. In this study, we aimed to identify the metastatic distribution patterns of first metastatic sites that significantly correlate with survival after recurrence.

**Methods:**

We performed a retrospective review of records from 271 patients with recurrent metastatic HR+/HER2- breast cancer diagnosed between January 2000 and December 2015. We assessed survival after recurrence according to the metastatic distribution patterns of the first metastatic sites and identified significant prognostic factors among patients with single and multiple metastases.

**Results:**

Prognosis was significantly better in patients with a single metastasis than in those with multiple metastases (median overall survival after recurrence: 5.86 years vs. 2.50 years, respectively, *p* < 0.001). No metastatic organ site with single metastasis was significantly associated with prognostic outcome, although single metastasis with diffuse lesions was an independent risk factor for worse prognosis (HR: 3.641; 95% CI: 1.856–7.141) and more easily progressing to multiple metastases (*p* = 0.002). Multiple metastases, including liver metastasis (HR: 3.145; 95% CI: 1.802–5.495) or brain metastasis (HR: 3.289; 95% CI: 1.355–7.937), were regarded as significant independent poor prognostic factors; however, multiple metastases not involving liver or brain metastasis were not significantly related to prognosis after recurrence.

**Conclusions:**

Single metastases with diffuse lesions could more easily disseminate systemically and progress to multiple metastases, leading to a poor prognosis similar to multiple metastases. Our findings indicate that the reconsideration of the determinant factors of therapeutic strategies for first recurrence in HR+/HER2- breast cancer may be needed.

## Introduction

Breast cancer is the most commonly diagnosed cancer in women and a leading cause of cancer-related mortality worldwide [1, 2]. Despite recent improvements in adjuvant treatment, 20–30% of early breast cancer patients will develop distant metastasis and be generally incurable, in which cases the main treatment goal is symptom palliation [3–5]. More than 70% of patients present with hormone receptor-positive (HR+) and human epidermal growth factor receptor 2-negative (HER2-) breast cancer [6]. According to the current guidelines and international consensus statements, the metastatic patterns of first metastatic sites are important determinants of the initial therapeutic strategy for HR+/HER2- metastatic patients [7–9]. However, the metastatic patterns of the first metastatic sites, such as multiple metastases, immediately life-threatening disease, or rapidly progressive visceral metastasis or crisis, have not been clearly evaluated. Although previous real-world studies have demonstrated several significant prognostic factors of survival after recurrence [10–24], little attention has been given to the influence of the metastatic distribution patterns of first metastatic sites on patient prognosis. We felt that the metastatic distribution patterns should be analyzed in more detail to evaluate the relationship with survival after recurrence in HR+/HER2- patients. Therefore, we used the clinical data of HR+/HER2- recurrent breast cancer patients with distant metastasis to identify the correlation between the metastatic distribution patterns of first metastatic sites and survival after recurrence. We evaluated the prognosis of each HR+/HER2- patient with single and multiple metastases separately. We also classified single metastases into diffuse lesions and non-diffuse lesions, which may help us determine more beneficial therapeutic strategies and meet the unmet needs of these patients. A deeper understanding of the metastatic distribution pattern in HR+/HER2- patients may be needed for the delivery of appropriate healthcare to poor-prognosis groups and play an important role in optimal treatment and care, thereby improving the prognosis of these patients.

## Methods

### Study design and patients

This study was conducted using a retrospective longitudinal cohort design with the use of hospital electronic patient records. Patients with a first diagnosis of recurrent HR+/HER2- breast cancer made between January 2000 and December 2015 were identified from Sakai City Medical Center and Kindai University Hospital. Patients treated within a clinical trial (prior to or during the study period) were excluded. The index date was that of first diagnosis of distant recurrent breast cancer. Follow-up was defined as the interval between the index date and the confirmed date of death, the censored date (if lost to follow-up) or the study end in December 2018. According to current guidelines, all patients received standard adjuvant treatment and were followed up with through a regular physical examination 1–4 times a year and annual mammography; if necessary, blood exams, ultrasonography, computed tomography (CT), bone scintigraphy, magnetic resonance imaging, or position emission tomography/CT were added for the diagnosis of recurrence. Recurrence was defined as the occurrence of distant metastasis after removal of the primary breast cancer. Patients with only locoregional recurrence and distant metastasis at initial diagnosis (de novo Stage IV metastatic disease) were excluded from this analysis. Ipsilateral breast tumor recurrence and ipsilateral axillary, infraclavicular, internal mammary, and supraclavicular lymph node metastasis were defined as locoregional recurrence. TNM staging was based on the criteria of the 8th Union for International Cancer Control. The adjuvant and metastatic treatment strategies (treatment protocol after recurrence) were all decided at the experts’ conference in the institutions based on current guidelines. This study was approved by the institutional review board of the two hospitals, and all enrolled patients provided informed consent.

### Immunohistochemical and serological assay

Positivity for estrogen receptor (ER) or progesterone receptor (PR) was defined as a score ≥ 3 using the Allread scoring system [25]. HR positivity was defined as ER and/or PR positivity. HER2 negativity was defined as an immunohistochemistry score of 0, 1+, or 2+ and negative fluorescence in situ hybridization (ratio < 2.0). The concentrations of serum carcinoembryonic antigen (CEA) and cancer antigen 15–3 (CA15–3) were measured at the first distant recurrence using an electrochemiluminescent immunoenzymometric assay (Roche Diagnostics, Tokyo, Japan). The upper limits of normal for CEA and CA15–3 were 5 ng/ml and 25 U/ml, respectively.

### Metastatic distribution patterns of first metastatic sites

The first metastatic sites were classified into single metastases or multiple metastases. Single metastases were further classified into diffuse or non-diffuse lesions. Non-diffuse lesions were defined as localized or focal lesions in a single metastatic organ or site regardless of size, whereas diffuse lesions were defined as multiple lesions widely spreading in a single metastatic organ or site. Non-diffuse lesions included a solitary metastatic lesion in one single organ (e.g., a solitary lung metastasis), the involvement of a single lymphatic site (e.g., an ipsilateral hilar lymph node metastasis), or a solitary or isolated metastatic bone lesion. On the other hand, diffuse lesions included multiple lesions in one single organ (e.g., multiple lung metastases), the involvement of two or more lymphatic sites (e.g., bilateral hilar lymph node metastases), multiple metastatic bone lesions, or pleural or peritoneal dissemination.

### Survival outcomes

Overall survival (OS) was defined as the time from the date of the first distant recurrence to the time of death or last follow-up. The disease-free interval (DFI) was defined as the interval between the diagnosis of primary nonmetastatic breast cancer and the date of the first distant recurrence. Time to multiple metastases (TTM) was defined as the time from the date of the first distant recurrence at a single metastatic site to the date of the progression of disease at multiple metastatic sites.

### Statistical analysis

OS plots were created using the Kaplan-Meier method, and the distributions of the survival curves were compared using log-rank tests. The Cox proportional hazard regression model was used to examine the prognostic evaluation between groups using several prognostic indicators, including patient and disease-related clinicopathological factors and metastatic organ sites and distribution patterns. A 95% confidence interval (CI) was calculated for all hazard ratios (HRs) in the Cox regression analysis. We evaluated the results of the univariate and multivariate Cox proportional hazards models with hazards ratios > 1.0 indicating an increased risk of death. All tests were two-tailed, and *p*-values < 0.05 were considered significant. Statistical analyses were performed using the statistical software package SPSS (v.17.0; Chicago, IL, USA).

## Results

### Patient characteristics

Our analysis included 271 patients with recurrent metastatic HR+/HER2- breast cancer during the study period. The median follow-up for our sample was 8.57 years (range, 1.05–19.67). The patient characteristics for the study cohort are summarized in Table 1. The median age at recurrence was 62 years (range, 29–92). The majority of the sample consisted of patients with single metastasis (*n* = 169, 62%), and bone was the most common metastatic organ site (*n* = 148, 55%) of all patients.

**Table 1:** Patient characteristics

Characteristics	All	Single metastasis			Multiple metastases
Number	271	169 (62%)			102 (38%)
Stage at diagnosis					
I	37 (14%)	24 (14%)			13 (13%)
IIA + IIB	84 + 89 (64%)	58 + 53 (66%)			62 (61%)
IIIA+IIIB+IIIC	32 + 22 + 7 (22%)	18 + 12 + 4 (20%)			27 (26%)
Adjuvant chemotherapy					
Yes	184 (68%)	115 (68%)			69 (68%)
No	87 (32%)	54 (32%)			33 (32%)
Median age at recurrence (range, years)	62 (29–92)	62 (29–90)			63 (33–92)
Median OS after recurrence (range, years)	4.58 (0.02–13.53)	5.86 (0.04–13.53)			2.50 (0.02–12.21)
DFI (median, years)	4.01 (0.35–16.85)	3.98 (0.58–12.02)			4.04 (0.35–16.85)
< 2 years	56 (21%)	35 (21%)			21 (21%)
≥2 years	215 (79%)	134 (79%)			81 (79%)
CEA/CA15–3 serum level					
Normal	106 (47%)	81 (57%)			25 (30%)
High	120 (53%)	62 (43%)			58 (70%)
Initial therapy for reccurence					
Endocrine therapy	173 (64%)	119 (70%)			54 (53%)
Chemotherapy	83 (31%)	42 (25%)			41 (40%)
unknown	15 (5%)	8 (5%)			7 (7%)
Metastatic organ site		All	Diffuse lesions	Non-diffuse lesions	
All	271	169	90 (53%)	79 (47%)	102
Bone	148 (55%)	85 (50%)	46 (54%)	39 (46%)	63 (62%)
Lymph node	95 (35%)	31 (18%)	13 (42%)	18 (58%)	64 (63%)
Lung	74 (27%)	24 (14%)	17 (71%)	7 (29%)	50 (49%)
Liver	54 (20%)	14 (8%)	7 (50%)	7 (50%)	40 (39%)
Pleural	41 (15%)	12 (7%)	6 (50%)	6 (50%)	29 (28%)
Brain	8 (3%)	1 (1%)	1 (100%)	0 (0%)	7 (7%)
Others	2 (1%)	2 (1%)	0 (0%)	2 (100%)	0 (0%)

### Survival outcomes

**Fig. 1:**
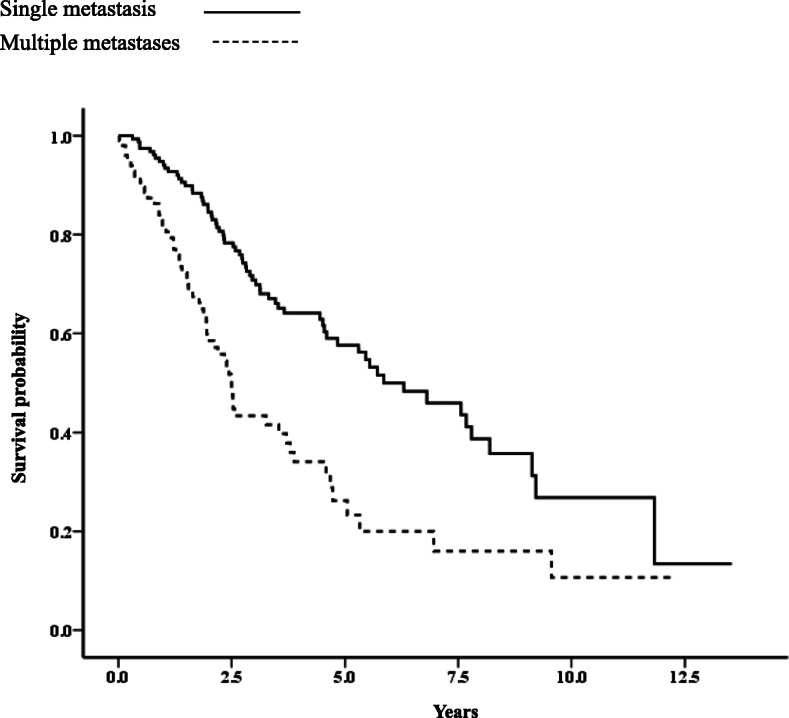
Kaplan-Meier curve for survival after recurrence between single and multiple metastases (*p* < 0.001)

**Fig. 2:**
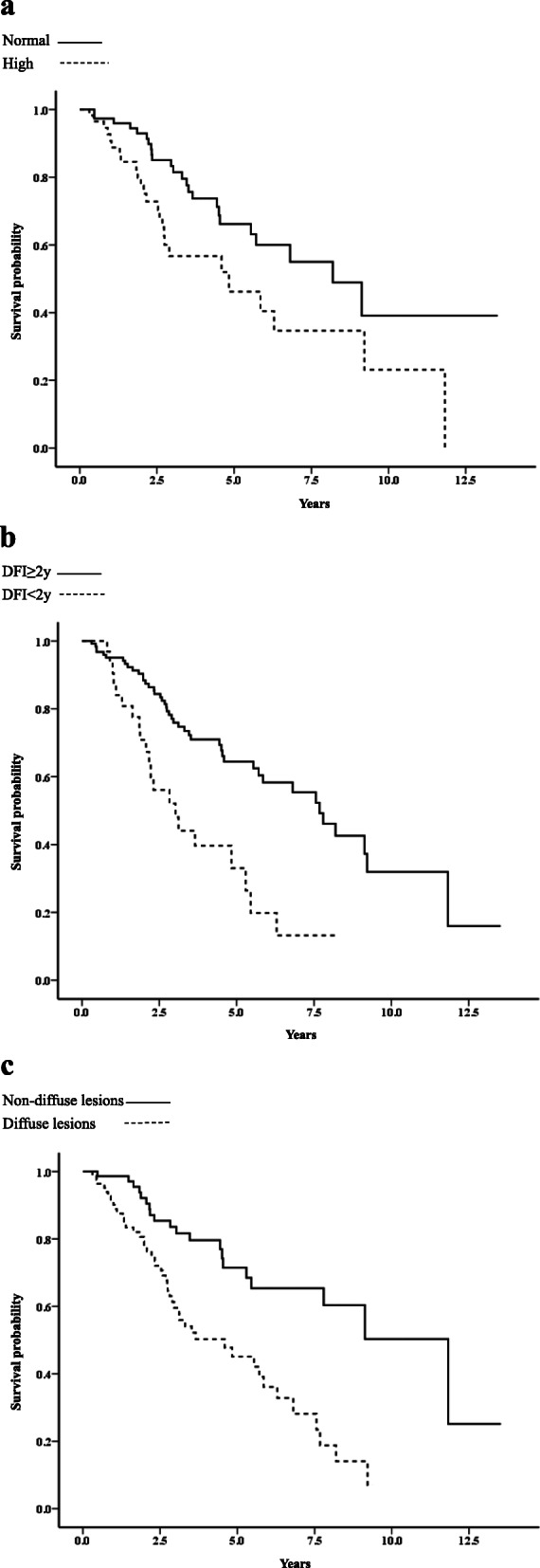
Kaplan-Meier curves for survival after recurrence according to **a** serum CEA/CA15–3 levels in single metastasis (*p* = 0.014), **b** DFI < 2 years and DFI ≥ 2 years in single metastasis (*p* < 0.001), and **c** diffuse lesions and non-diffuse lesions in single metastasis (*p* < 0.001)

The median OS after recurrence estimated according to patient characteristics is given in Table 2. The median OS after recurrence for patients with single metastasis and multiple metastases was 5.86 and 2.50 years, respectively (*p* < 0.001; Fig. 1). Of the patients with a single metastasis, those with high serum levels of CEA/CA15–3 (*p* = 0.014; Fig. 2a), shorter DFI (< 2 years, *p* < 0.001; Fig. 2b), or diffuse lesions had significantly worse prognosis (*p* < 0.001; Fig. 2c), whereas any metastatic organ site with a single metastasis was not significantly associated with prognostic outcomes. Of the patients with multiple metastases, patients with shorter DFI, liver metastasis, or brain metastasis had significantly worse prognosis. However, multiple metastases not involving liver or brain metastasis had no significant relationship with prognostic outcomes.

**Table 2:** Median overall survival (OS) after recurrence according to patient characteristics

Characteristics	All		Single metastasis		Multiple metastases	
	Median OS (year)	*p*-value	Median OS (year)	*p*-value	Median OS (year)	*p*-value
All patients	4.58		5.86		2.50	< 0.001
Stage at diagnosis		0.195		0.840		0.073
I + II	4.60		6.30		2.53	
III	3.79		5.30		1.78	
Adjuvant chemotherapy		0.446		0.149		0.613
Yes	4.44		5.46		2.50	
No	5.55		7.56		2.35	
Age at recurrence		0.972		0.259		0.596
< 50y	3.52		4.54		2.39	
≥ 50y	4.67		6.81		2.50	
DFI		< 0.001		< 0.001		< 0.001
< 2 years	2.17		3.03		1.35	
≥ 2 years	5.33		7.68		3.26	
CEA/CA15–3 serum level		< 0.001		0.014		0.390
Normal	8.20		8.20		3.54	
High	3.26		4.83		2.49	
Initial therapy for reccurence		0.077		0.348		0.524
Endocrine therapy	5.46		6.30		2.53	
Chemotherapy	3.12		5.30		2.50	
Distribution pattern in single metastasis				< 0.001		
Diffuse lesions	–		4.60		–	
Non-diffuse lesions	–		11.83		–	
Metastatic organ site						
Bone	4.51 (vs. 4.83)	0.443	5.86 (vs. 6.30)	0.869	1.96 (vs. 3.26)	0.327
Lymph node	3.54 (vs. 5.46)	0.022	4.54 (vs. 7.56)	0.088	2.57 (vs. 2.20)	0.279
Lung	5.72 (vs. 4.54)	0.688	8.20 (vs. 5.46)	0.062	2.50 (vs. 2.44)	0.287
Liver	1.96 (vs. 5.30)	< 0.001	9.13 (vs. 5.72)	0.670	1.88 (vs. 3.87)	< 0.001
Pleura	3.54 (vs. 4.73)	0.204	2.90 (vs. 6.30)	0.153	3.54 (vs. 2.39)	0.254
Brain	0.800 (vs. 4.67)	< 0.001	–	–	0.800 (vs. 2.50)	0.027

### Univariate and multivariate analysis of factors related to survival after recurrence

Multivariate analysis was performed according to the prognostic factors that were significant in the univariate analysis in patients with single or multiple metastases. The multivariate analysis revealed two significantly independent prognostic factors related to poor survival after recurrence in patients with single metastasis: shorter DFI and diffuse lesions (Table 3). The multivariate analysis revealed three significantly independent prognostic factors related to poor survival after recurrence in patients with multiple metastases: shorter DFI, liver metastasis, and brain metastasis (Table 4).

**Table 3:** Univariate and multivariate analyses in patients with single metastatasis

Characteristics	Single metastasis			
	Univariate analysis		Multivariate analysis	
	HRs (95%CI)	*p*-value	HRs (95%CI)	*p*-value
Stage at diagnosis				
I + II	1.00	0.840		
III	1.068 (0.564–2.024)			
Adjuvant chemotherapy				
Yes	1.00	0.152		
No	0.661 (0.375–1.165)			
Age at recurrence				
< 50y	1.00			
≥ 50y	0.726 (0.415–1.270)	0.261		
CEA/CA15–3 serum level				
Normal	1.00			
High	2.001 (1.138–3.517)	0.016	1.543 (0.866–2.750)	0.141
DFI				
< 2 years	2.808 (1.643–4.797)	< 0.001	3.527 (1.891–6.576)	< 0.001
≥ 2 years	1.00			
Initial therapy for reccurence				
Endocrine therapy	1.00	0.349		
Chemotherapy	1.285 (0.761–2.170)			
Distribution pattern				
Diffuse lesions	2.922 (1.699–5.025)	< 0.001	3.641 (1.856–7.141)	< 0.001
Non-diffuse lesions	1.00			
Metastatic organ site				
Bone	0.960 (0.589–1.565)	0.869		
Lymph node	1.678 (0.919–3.067)	0.092		
Lung	0.497 (0.235–1.050)	0.067		
Liver	1.221 (0.488–3.055)	0.670		
Pleura	1.927 (0.770–4.831)	0.161		
Brain	–	–

**Table 4:** Univariate and multivariate analyses in patients with multiple metastases

Characteristics	Multiple metastases			
	Univariate analysis		Multivariate analysis	
	HRs (95%CI)	*p*-value	HRs (95%CI)	*p*-value
Stage at diagnosis				
I + II	1.00	0.076		
III	1.695 (0.946–3.030)			
Adjuvant chemotherapy				
Yes	1.00	0.613		
No	1.151 (0.667–1.989)			
Age at recurrence				
< 50y	1.00			
≥ 50y	0.807 (0.366–1.782)	0.596		
CEA/CA15–3 serum level				
Normal	1.00			
High	1.337 (0.688–2.595)	0.391		
DFI				
< 2 years	3.229 (1.768–5.895)	< 0.001	3.082 (1.669–5.694)	< 0.001
≥ 2 years	1.00			
Initial therapy for reccurence				
Endocrine therapy	1.00	0.524		
Chemotherapy	1.187 (0.700–2.013)			
Metastatic organ site				
Bone	0.769 (0.455–1.301)	0.328		
Lymph node	1.334 (0.790–2.255)	0.281		
Lung	0.757 (0.452–1.266)	0.289		
Liver	2.915 (1.689–5.025)	< 0.001	3.145 (1.802–5.495)	< 0.001
Pleura	1.406 (0.781–2.530)	0.256		
Brain	2.532 (1.078–5.952)	0.033	3.289 (1.355–7.937)	0.008

### TTM for diffuse lesions and non-diffuse lesions

**Fig. 3:**
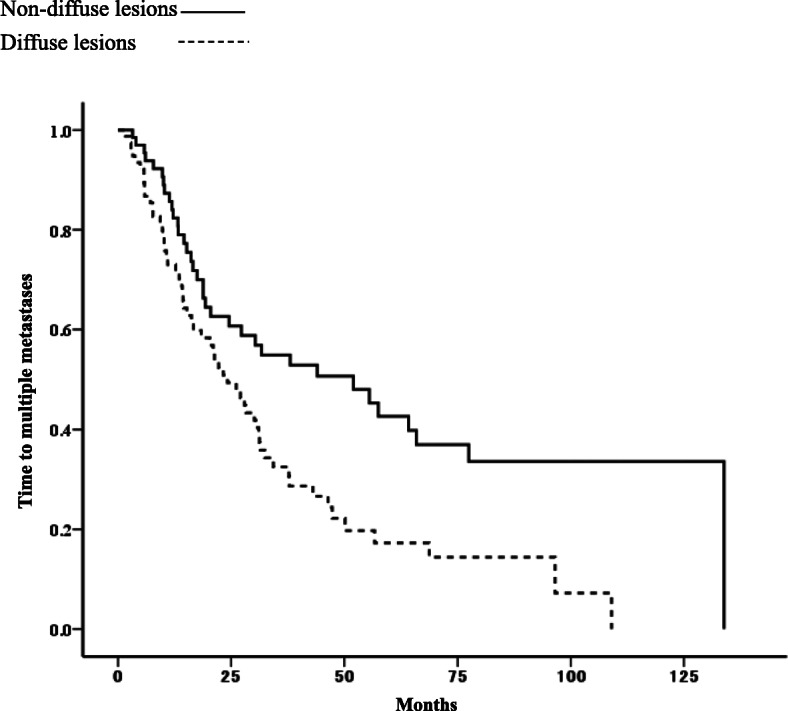
Time to multiple metastases in patients with diffuse lesions and non-diffuse lesions in single metastasis (*p* = 0.002)

Survival plots showed that TTM was significantly shorter for patients with diffuse lesions among those with single metastasis than for those with non-diffuse lesions (median TTM: 24.2 months vs. 52.0 months, *p* = 0.002; Fig. 3).

## Discussion

Metastatic breast cancer represents a biologically heterogeneous population with diverse metastatic patterns exhibiting highly unpredictable clinical behaviors [26–28]. Prognosis varies significantly among patient subtypes [29, 30], and several concepts regarding the prognosis of HR+/HER2- metastatic patients have been considered important in evaluating survival after recurrence [11]. Previous studies have shown that first metastatic sites (single, multiple, liver, or other visceral metastases) are significantly related to survival after recurrence in HR+/HER2- patients [10–13]; however, there has been clinical diversity in the metastatic distribution patterns of both single metastasis and multiple metastases [26–28]. Therefore, we studied the prognosis of HR+/HER2- patients with single and multiple metastases separately, with emphasis on the metastatic distribution patterns.

Multiple metastases are considered a poor prognostic factor [10–18]. The prognosis of patients with multiple metastases may vary with the metastatic organ sites due to the heterogeneity [26–28], but there has been no report investigating prognosis by each relevant metastatic organ site involved in multiple metastases. In the current study, multiple metastases including the liver or brain were a strong independent prognostic factor for worse outcomes in HR+/HER2- patients, whereas multiple metastases not involving the liver or brain had no significant relationship with prognosis after recurrence. This finding supports the assumption that multiple metastases involving liver or brain metastasis are indicative of extensive spreading, dissemination of cancer cells, or lethal organ dysfunction, leading to poor survival outcomes [11, 15, 24]. In real-world practice, patients with multiple metastases are more likely to receive cytotoxic therapy because their vital organs are potentially damaged or in “visceral crisis” [19–21]. However, due to their own heterogeneity, not all multiple metastases may lead to poor outcomes [19]; therefore, we should determine the most reliable and decisive prognostic factors for HR+/HER2- patients with multiple metastases.

Single metastasis has been regarded as a better prognostic factor than multiple metastases due to the probability of less tumor burden [10–18]. However, most patients with a single metastasis will eventually develop multiple metastases, eventually leading to poor outcomes. In this study, we analyzed the prognosis of patients with single metastases by classifying single metastases into diffuse and non-diffuse lesions. To the best of our knowledge, this is the first study to investigate the prognosis of patients with single metastases based on the metastatic distribution patterns between diffuse and non-diffuse lesions, and to evaluate the time to dissemination of single metastases to multiple metastases. We found that diffuse lesions in a single metastasis were independently related to worse prognosis and easier systemic dissemination into multiple metastases. Diffuse lesions in single metastases may likely behave as multiple metastases due to the dissemination potential. According to traditional guidelines [7–9], noncytotoxic therapy is indicated as a first-line treatment for HR+/HER2- patients with a single metastasis. Thus, our study suggests reconsidering the therapeutic guidelines, and additional treatment strategies should be sought for patients with diffuse lesions in single metastases. Our proposal may meet the unmet need for more efficacious treatments for HR+/HER2- patients with diffuse lesions in a single metastasis. The initial use of more advantageous treatments, including novel targeted agents [31], could provide more beneficial effects and better prognoses for these patients.

Our study had some limitations. First, our study was performed as a retrospective chart review without validation, and sampling biases may have been unavoidable. Second, the sample size of our study was small, and our results should be interpreted with caution. However, the selection of patients with HR+/HER2- recurrent breast cancer and exclusion of HER2+, triple-negative, and de novo breast cancer patients may have allowed the recruitment of a patient population with relative homogeneity. Future studies with a larger cohort of patients may yield more conclusive evidence of the prognostic findings in this study. Despite these limitations, the current study with a uniform population provides important insights into the real-world clinical outcomes for patients with recurrent metastatic HR+/HER2- breast cancer.

## Conclusion

Our study presents new evidence of metastatic patterns and real-world clinical outcomes for HR+/HER2- recurrent metastatic breast cancer. We confirmed that multiple metastases not involving liver or brain metastasis had no significant relationship with prognosis after recurrence. We also confirmed that single metastasis with diffuse lesions was an independent factor for worse prognosis, with easier systemic dissemination and progression to multiple metastases than non-diffuse lesions. These findings may require reconsideration of the determinants of initial therapy for HR+/HER2- recurrent metastatic breast cancer and provide frontline physicians with new important clinical clues to achieve optimal treatment, leading to effective therapeutic strategies to improve the prognosis of this metastatic disease.

## Data Availability

The datasets used and analyzed during this study are available from the corresponding author on reasonable request.

## Abbreviations

HR: Hormone receptor-positive (HR+)
HER2-: Human epidermal growth factor receptor 2-negative
CT: Computed tomography
ER: Estrogen receptor
PR: Progesterone receptor
CEA: Carcinoembryonic antigen
CA15–3: Cancer antigen 15–3
OS: Overall survival
DFI: Disease-free interval
TTM: Time to multiple metastases
CI: Confidence interval
HRs: Hazard ratios
